# Chitinase of *Trichoderma longibrachiatum* for control of *Aphis gossypii* in cotton plants

**DOI:** 10.1038/s41598-023-39965-y

**Published:** 2023-08-14

**Authors:** Waheed Anwar, Huma Amin, Hafiz Azhar Ali Khan, Adnan Akhter, Uzma Bashir, Tehmina Anjum, Rabia Kalsoom, Muhammad Asim Javed, Karamat Ali Zohaib

**Affiliations:** 1 Department of Plant Pathology, Faculty of Agricultural Sciences, University of the Punjab, Lahore, Pakistan; 2 Department of Entomology, Faculty of Agricultural Sciences, University of the Punjab, Lahore, Pakistan; 3 Institute of Zoology, Faculty of Life Sciences, University of the Punjab, Lahore, Pakistan

**Keywords:** Plant biotechnology, Genetic engineering

## Abstract

Chitinase-producing fungi have now engrossed attention as one of the potential agents for the control of insect pests. Entomopathogenic fungi are used in different regions of the world to control economically important insects. However, the role of fungal chitinases are not well studied in their infection mechanism to insects. In this study, Chitinase of entomopathogenic fungi *Trichoderma longibrachiatum* was evaluated to control *Aphis gossypii.* For this purpose, fungal chitinase (*Chit1*) gene from the genomic DNA of *T. longibrachiatum* were isolated, amplified and characterised. Genomic analysis of the amplified *Chit1* showed that this gene has homology to family 18 of glycosyl hydrolyses. Further, *Chit1* was expressed in the cotton plant for transient expression through the Geminivirus-mediated gene silencing vector derived from Cotton Leaf Crumple Virus (CLCrV). Transformed cotton plants showed greater chitinase activity than control, and they were resistant against nymphs and adults of *A. gossypii.* About 38.75% and 21.67% mortality of both nymphs and adults, respectively, were observed by using *Chit1* of *T. longibrachiatum*. It is concluded that *T. longibrachiatum* showed promising results in controlling aphids by producing fungal chitinase in cotton plants and could be used as an effective method in the future.

## Introduction

Crops are vulnerable to attack by many biotic factors including phytopathogens^1^. Mostly, insect pests not only involved in yield losses but also affect the overall quality and quantity of agricultural products^2^. Aphids are the most devastating economical pest of various field, ornamental and greenhouse crops such as tomato, cucumber, pepper, cotton, wheat, brassica and rose^3–5^. Cotton (*Gossypium hirsutum *L.) is an important cash crop and is highly susceptible to attack by aphids^6^. Cotton aphids (*Aphis gossypii*), as a vicious pest of cotton that usually suck the phloem sap from vascular plant parts of cotton and help in the transmission mechanism of more than 75 viruses^7,8^. Major damages caused by *A. gossypii* are associated with its remarkable deposition of sooty mold and secretion of honeydews on cotton balls^9,10^. Cotton aphids areone of the notorious pests due to viviparous in nature and has high production rates in a short interval of oviposition periods^9^.

In the past, aphids were usually managed using various insecticides in both greenhouse and field crops; but it has resulted in severe socio-economical, environmental and health problems^10^. The high and injudicious uses of chemical insecticides have enhanced the resurgence and resistance of most insect pests. Besides this, the high input cost of insecticides and all legal restrictions have focused on using an alternative approach to control insect pests^11,12^. In the previous years, an alternative bio-control method is in specifically, the use of entomopathogens has emerged^13,14^. Entomopathogenic fungi [EPF] have exclusive characterisations that enable them to be the potential bio-control agents of insect pests. Due to their vast host range, almost 1000 different species of EPF are involved in effectively controlling many insects species^15,16^. Various insect species such as thrips, whiteflies, aphids, mealybugs, psyllids, weevils and nematodes are hosts of EPF in both field and greenhouses^17–19^. Different species and strains of *Trichoderma* like *T. afroharzianum* and *T. atroviride* are actively found in the bio-control of aphids at their different morphological stages. Studies carried out to date have reported that *Trichoderma* is capable of controlling insect pests directly through parasitism and by the production of insecticidal secondary metabolites, antifeedant compounds and repellent metabolites. While, indirectly through the activation of systemic plant defensive responses, the attraction of natural enemies or the parasitism of insect-symbiotic microorganisms. It has been shown that *Trichoderma* may also have complementary properties that enhance plant defense barriers against insect pests^20,21^. However, it is not easy to apply entomopathogens under field conditions along with other management practices due to differences in their mode of action^22^. Currently, scientists are applying biotechnological techniques to find unique genes that can be incorporated into the genome of the host plant to create resistance against specific insect pests. Over the past few years, various studies have highlighted the secretion of specific enzymes such as chitinases and proteases by EPF, which are playing a crucial role as insecticidal^23^. The outer cuticle of the insects is usually composed of chitin, embedded with a protein matrix. The extracellular enzymatic compounds produced by EPF can degrade these chitinous compounds and obtain nutrients from the insect’s hemolymph^24,25^. The fungal chitinases usually belong to class 3 chitinases and are considered an important part of the family 18 of glycosyl hydrolases^26^. Fungal chitinases of family 18 contain a chitin-binding domain that efficiently depolarises chitin^27,28^. The main structural component of an insect’s body is chitin that is a poly-β-1, 4-N-acetylglucosamine and almost 40% of the insect’s body mass is composed of it^29,30^. Taking into consideration, chitinase from the insect's associated fungi can be used to develop transgenic plants for the effective control of aphids. The overall expression of the *Chit1* gene in *T. longibrachiatum* is actively involved in enhancing the efficiency of insect cuticle digestion and enabling the increased virulence against insects. The role of various fungal secondary metabolites and extracellular enzymes is well documented to control the population of aphids^31,32^.

Genetic engineering techniques are employed to develop resistance in cotton plants against fungal pathogens, but there is lack of data related to the transgenic approach of chitinase genes against insect pests^33^. Various research studies are in progress about the transgenic expression of chitinases of fungus in cotton for transgenic plant development^34^. Therefore, the main objectives of our study were to isolate and characterise the chitinase gene from the fungal strain of *T. longibrachiatum,* development of transgenic cotton plants and analyse the overall resistance of transgenics against cotton aphids. The projected results will help to understand the use of transient chitinase plants for the bio-control of insects pests in the future.

## Results

### Isolation of endochitinase *Chit1*

**Figure 1:**
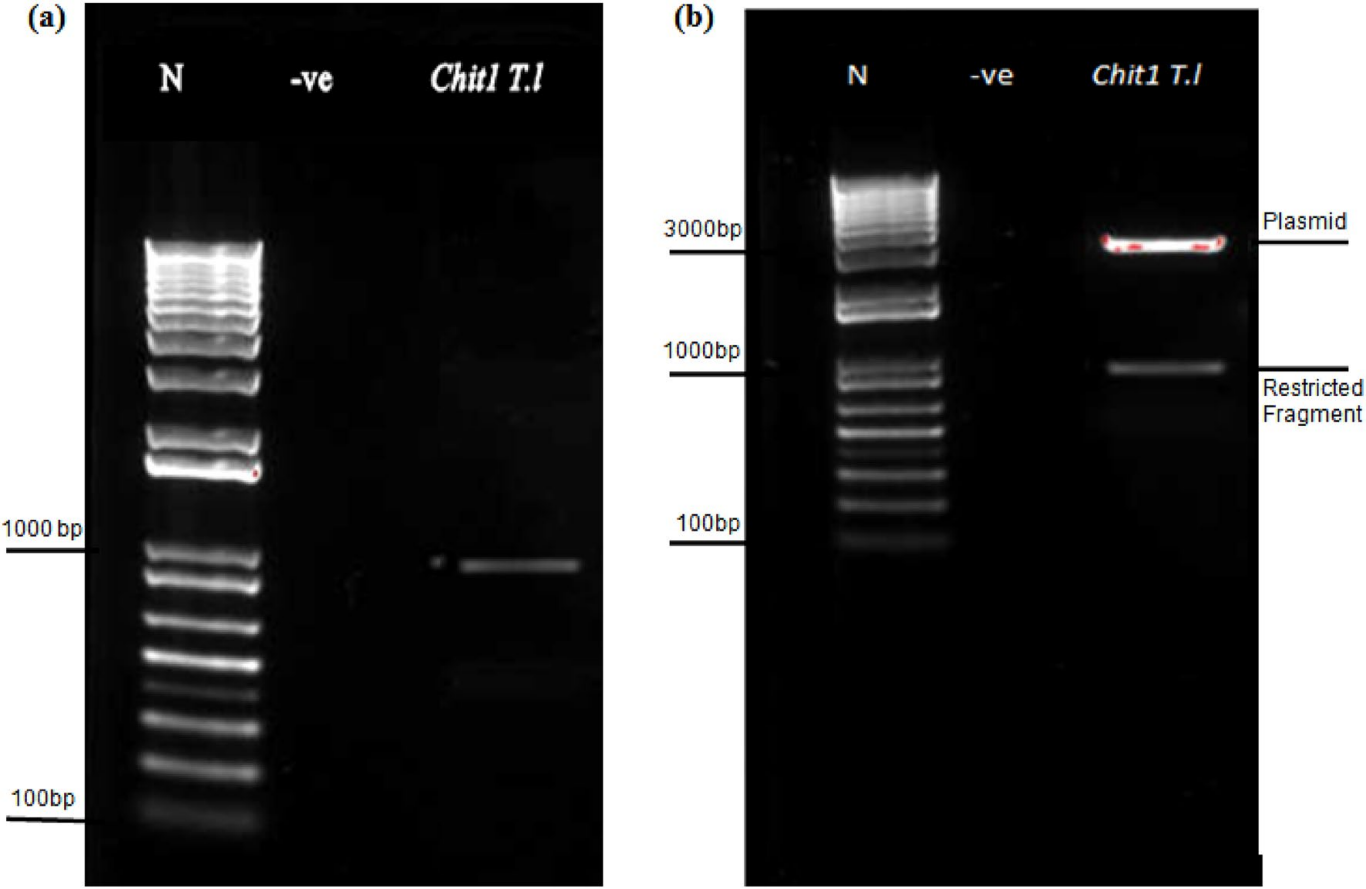
(**a**) PCR confirmation of partial endochitinase *Chit1* from *T. longibrachiatum* and (**b**) showed restriction analysis of recombinantby using *EcoR1* restriction enzyme. N represents PROMEGA plus 1 kb DNA ladder, -ve represent negative reaction and *Chit1* T.1 represent chitinase of *T. longibrachaitum.*

The chitinase gene was isolated from the genomic DNA of *T. longibrachiatum* and a product size of approximately 1 kb for *Chit1* is produced through PCR (Fig. 1a). The restriction digestion resulted in confirmation of partial recombinant clones of *Chit1* (Fig. 1b), and M13 primers were further used to sequence. The sequences of vector were removed from the nucleotide sequence using GENE TOOL and then subjected to BLAST analysis. The result confirms that the nucleotide sequences of *Chit1* had a maximum resemblance to endochitinases as compared to other EPF. *Chit1* showed remarkable homology of 99.52% to endochitinase from *T. longibrachiatum Chit1* (KJ787131).

### Characterisation of endochitinase *Chit1* from an isolate of *T. longibrachiatum*

The endochitinase gene of *T. longibrachiatum* was mainly composed of 216 amino acids.*.* The molecular mass of the encoded protein of *T. longibrachiatum* was 23.41 kDa, acidic in nature with a pI value of 4.5 (Table 1). In the case of *T. longibrachiatum*, the gene's sequence analyses expressed its origin. It was assessed that this gene has a domain belonging to catalytic glycosyl hydrolase family 18. The sequence alignment explained about the structural motif. The catalytic domain DGIDVDWEYP (DxxDxDxE) is dependent on the structural motif and is conserved in the chitinase gene. The substrate's binding site was at 6aa, while the active site of the catalyst was placed at site 43aa. The sequence was blasted, and it showed 99% similarity with the sequence of *T. longibrachiatum* strain ACD46738 and CDM98718.

**Table 1:** Molecular weight, pI value and sequences of the active sites of *T. longibrachiatum.*

Species	Molecular weight	pI value	Signal peptides	Sequences of active sites
*T. longibrachiatum*	23.41 kDa	4.5	–	DGIDVDWEYP

### Phylogenetic analysis of *T. longibrachiatum*

**Figure 2:**
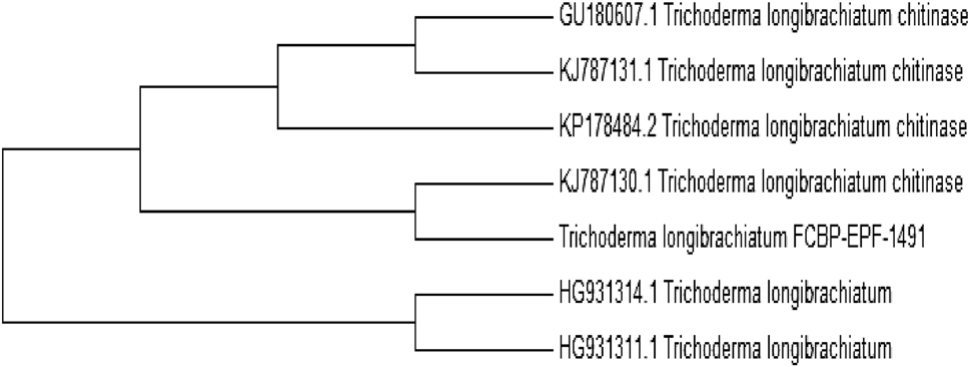
Phylogenetic analysis of endochitinase isolated from *Trichoderma longibrachiatum.*

The phylogenetic analysis was performed to find out the genetic variation among chitinases of different *Trichoderma* species*.* In phylogenetic tree analysis, endochitinase of fungal isolates were grouped into various clades and it showed maximum homologies to class 18 basic chitinases*. T. longibrachiatum* (Accession no: OQ689733) was classified into distant clades, and it showed maximum resemblance with *T. longibrachiatum Chit1* (KJ787131). Overall comparative sequence analysis exposed the fact that endochitinase *Chit1* is most related to the 18 glycosyl hydrolases family (Fig. 2).

### Chitinase confirmation in cotton plants

**Figure 3:**
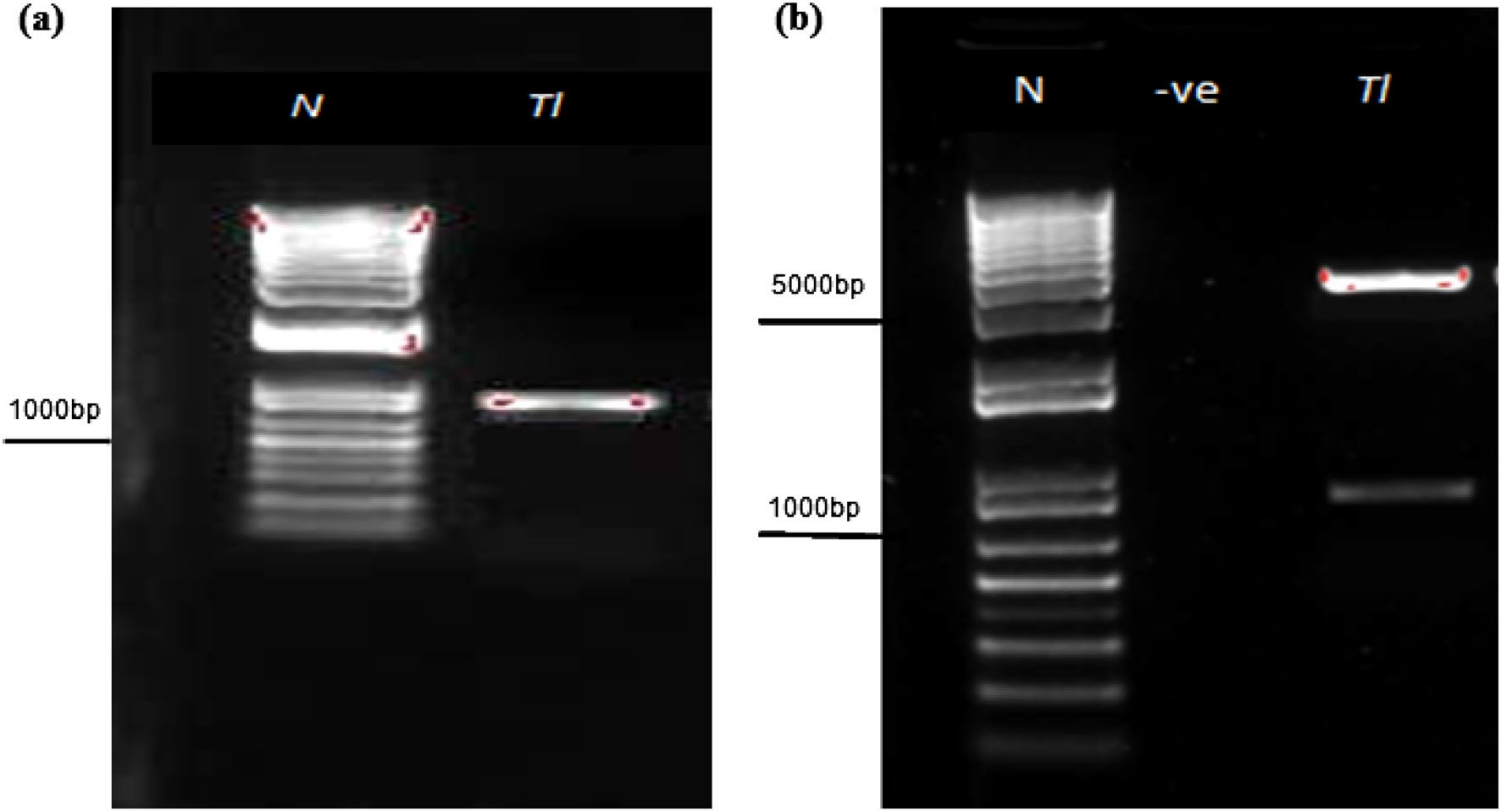
(**a**) Amplification of chitinase ORFs from *T. longibrachiatum* (**b**) Restriction analysis of *VIGS-Chit* recombinant plasmids. N represents PROMEGA1 kb Plus DNA ladder.

**Figure 4:**
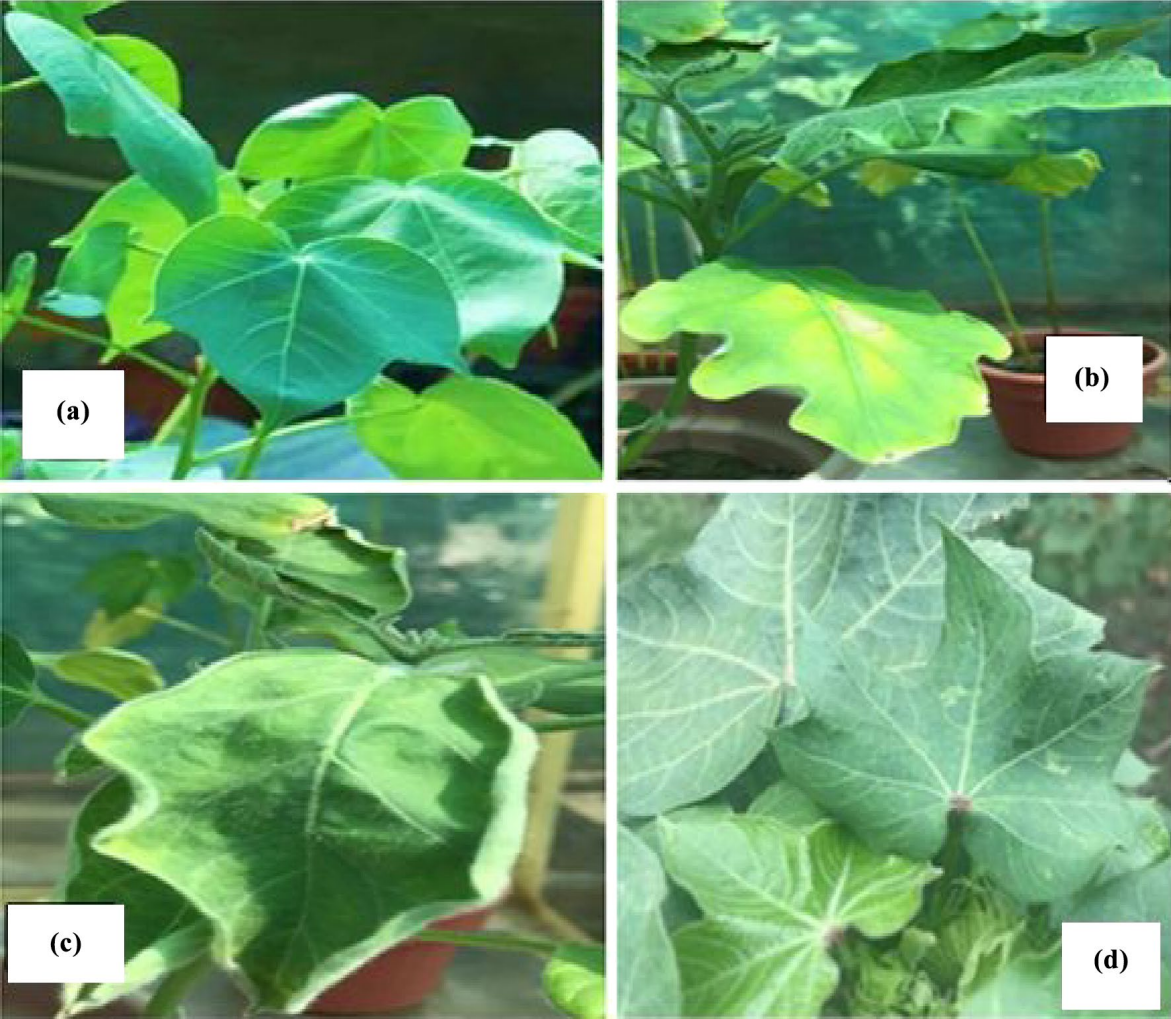
Visualisation of CLCrV, **(a)** control plant, **(b, c**) chitinase induced plants, (**d**) mock plant.

To confirm the chitinases in cotton plants, ORF the encoding conserved domain of catalytic family 18 of *Chit1* genes *T. longibrachiatum* was selected. The selection was made to detect the presence of the chitinases gene in cotton plants through a VIGS vector that is using a component of CLCrV. The amplicon of around 900 bp was observed from the amplification of recombinant plasmids using specific ORFs primers (Fig. 3a). VGS-*Chit1*_ *Tl* construct was confirmed by restriction analysis and sequencing (Fig. 3b). After 7 days of inoculation, amplification was positive for all plants. The development of different symptoms of CLCrV on cotton plants also revealed the expression of chitinase in plants as CLCrV is modified natural vector from Cotton Curl Crumple Virus and it shows similar symptoms in the expression of a particular gene (Fig. 4).

### Chitinase activity assays

The transformed cotton plants produced chitinase after 5 days. All the plants, including mock and control had produced chitinase, but chitinase production is higher in transformed plants than that of control plants. The transformed cotton plants of *Chit1* of *T. longibrachiatum* showed the chitinase activity of 5.64 U/mL. The overall difference in chitinase activity was found by reducing the chitinase gene from the control value. The chitinase activity of the control plant that had both VIGS and gene GUS was 5.09 U/mL, whereas chitinase activity was 5.44 U/mL in mock plants. The overall chitinase activity was shown in Table 2.

**Table 2:** Chitinase activity of transgenic cotton plants. n = 5.

Chitinase	Total chitinase activity of transient plants(U/ml)	Chitinase activity of control plant (U/ml)	Net Chitinase activity
*Chit1* Transgenic Plants	5.64a	5.09	0.55a
Mock Plants	5.44ab	5.09	0.35b

### Bioassays of transformed cotton plants against nymphs and adults of *A. gossypii*

Transformed cotton plants exhibited excellent virulence against the 4th nymphal instar and adults of *A. gossypii. Chit1* of *T. longibrachiatum* expressed in cotton plants showed promising results and the mortality of nymphs of about 38.75% after 144 h (Table 3). The highest adult mortality of 21.67% was recorded for *Chit1 T. longibrachiatum* transgenic plants (Table 3). The virulence efficiency of *T. longibrachiatum* can be increased by the overall production of chitinases, and the nymphal stage was more vulnerable to mortality compared to adults.

**Table 3:** Percentage mortality of the fourth instar and adults of *A. gossypii* against chitinase transgenic plants. Values with different letters showed significant difference at p < 0.05.

Mortality after		36 h	48 h	72 h	96 h	120 h	144 h
Control	Nymphs	–	–	–	–	–	8.25 ± 1.27e
*Chit1 T. longibrachiatum*	Nymphs	–	–	–	13.22 ± 1.00d	22.14 ± 1.47c	38.75 ± 1.91a
Control	Adults	–	–	–	–	–	5.54 ± 1.09fg
*Chit1 T. longibrachiatum*	Adults	–	–	–	6.32 ± 1.21ef	18.11 ± 1.80c	21.67 ± 2.09b

## Discussion

Entomopathogenic fungi are considered a potential microbial bio-control agent due to the production of various secondary metabolites and enzymes that act as insecticide and bio-pesticides. Plants are vulnerable to attack by pathogens, and usually, various resistance genes are encoded in plants to impart resistance. Chitinase genes canbecome effective now–a–day just like *Bacillus thuringiensis,* Bt gene was transfered to produce transgenic cotton in an effort to protectec the environment from overuse of pesticides^43^. It has been reported that microbial chitinases from many fungal strains were stable at broad ranges of temperature and pH, while showcased their effectiveness against graminaceous stem borers *Eldana saccharina* along withother lepidopteron pests^44^. *Chit1* was isolated from EPF (*T. longibrachiatum), *which belongs to family 18 of glycosyl hydrolases^45^. Insoluble chitin can bind specifically with chitinase using the chitin-binding domain^46^. Endochitinase genes from *T. harzianum* had been isolated and expressed in potatoes and tomatoes, and it had shown promising results without any adverse effect on the growth and development of both test plants^47^.

Our study was aimed to evaluate the transient expression of cotton plants and the overall efficiency of *Chit1* to control *A. gossypii*. The proper functioning of the transgenic product was analysed preliminary through the transference of the desired gene in the target cotton plant. Chitinase enzyme assays were performed to find out endochitinase activity, and transgenic plants with *T. longibrachiatum* (FCBP-EPF-1491) has exhibited chitinases activity of 0.55 U/mL after six days. While, previously, chitinase activity of 0.585 U/mL was recorded after 4 days of incubation^48^. The highest chitinase activity after 3 to 4 day of fungus production on SDAY(Sabouraud dextrose agar yeast) media was observed. The activity was 6.96 to 46.49 U/mL after 2 h of fungus incubation with 10% colloidal chitin at 37 ºC^49^. In our study, the chitinase activity of transgenic plants with *Chit1* of *T. longibrachiatum* was recorded as 0.55 U/mL. Chitinase production by *T. longibrachiatum* was observed^50^*,* and maximum production was 6.45 U/mL in plants.Various species of *Trichoderma* produce enzymes of different concentrations depending upon the substrates. In vitro conditions, various species such as *T. gamsii, T. virens*, *T. longibrachiatum* and *T. asperellum* produced maximum chitinase enzyme of about 0.151 U/mL, 0.107 U/mL, 0.228 U/mL and 0.164 U/mL, respectively. Above all, *T. longibrachiatum* produced a maximum chitinase of 0.228 U/mL under optimal provided conditions^51^. Production of chitinase varied according to the substrates, pH and temperature. The different isolates of the same fungal strain can produce chitinases in various concentrations. The activity of chitinase production usually alters by changing the incubation period. But, incubation exceeding six days reduced the amount of chitinase enzyme. The chitinolytic activity of most of fungal strains enhanced at the pH range of 4.0 to 5.2^52^. This study explained the overall production of fungal isolate and difference in production of enzymes in both control and inoculated plants.

Chitinases in the plants are usually involved in the safety of plants against damaging plant pathogens. Most of the induced gene plants showed mild symptoms that confirm the successful transformation of the desired gene in plants. In CLCrV, the plants usually produced light colour veins and downward curling of cotton leaves that confirm the difference between transformed and non-transformed cotton plants. In the case of Virus-induced gene silencing in two model plants that are *Arabidopsis* and *N. benthamiana*, severe development of symptoms were displayed when compared to the tobacco rattle virus^53,54^. Our result documented that transgenic plants with *Chit1* of *T. longibrachiatum* recorded the mortality of nymphs of about 38.75% after 144 h of insect feeding. The mortality of adults of *A. gossypii* started after 120 h of feeding on transformed cotton plants. The maximum adult mortality of 21.67% was recorded for *Chit1* trangenic cotton*.*

The use of chitinase has revolutionised the agricultural sector as it has altered the use of chemical pesticides. Previously, the chitinase from *T. longibrachiatum* reported to be widely used to reduce the population of root-knot nematode (*Meloidogyne javanica*)^55^*.* Fungal enzymes and culture filtrate of *Metarhizium anisopliae* and *T. harzianum* were toxic to cotton bollworms and mosquitos^56,57^. The difference in mortality percentage and efficacy of bio-control usually depends on the use of fungal strains and species of insects. In the future, it is highly recommended to use synergistic genes to enhance the overall efficiency of bio-control. It is also recommended to meditate on RNAi (RNA interference) to control the most economical agricultural pests.

## Conclusion

In conclusion, fungal chitinases can be widely used to control different agricultural pests, including insects and nematodes, at various morphological states. The cotton plant showed great flexibility for the incorporation of *Chit1* genes of *T. longibrachiatum. Chit1* showed promising results to control *A. gossypii*. The formulations of fungal chitinases are less in use as compared to *Bt* formulations. So, there is a huge need to work on formulations, effect of all possible fungal strains and their effects on insect pests to use as bio-control agents at a vast scale to preserve the environment from the lethal and sublethal effects of pesticides. In future, more chitinase gene can be isolated from different entomopathogenic fungi that can be used to enhance the virulence of transgenic cotton.

## Materials and methods

### Isolation of the chitinase gene from *Trichoderma longibrachiatum*

The fungal culture of *T. longibrachiatum* was obtained from the First Fungal Culture Bank of Pakistan (FCBP), Faculty of Agricultural Sciences, University of the Punjab, Lahore, Pakistan. The chitinase gene was amplified from fungal strains of *T. longibrachiatum* (FCBP-EPF-1491) that were initially isolated from whitefly^35^. The genomic DNAwas extracted by using the Cetyl Trimethyl Ammonium Bromide (CTAB) buffer method36. The whitefly DNA was extracted in preheated (65 °C) CTAB buffer. The solution was then mixed with chloroform and centrifuged (16,000 rpm for 4 min) to collect the supernant. The supernatant mixed with isopropanol, and again centrifuged (16,000 rpm for 8 min), followed by washing with ethanol (70%). PCR of samples was performed by using primers; *Tri_Chit1_*F (5′GCATCTGTGATTTTGCATAC3′) and *Tri_Chit1_*R **(′**5′GCCAAGAGACTTGAGGTAAG3′)^35^. The reaction mixture for PCRwas prepared, that had 18.4 μL of nuclease-free water, 1 μL of 10 mM dNTPs, 0.1 μL of Taq polymerase (5 U μL^−1^, THERMO FISHER SCIENTIFIC), 0.5 μL of 25 mMMgCl_2,_ 2 μL of 1X tag buffer, 0.5 μL of forward and reverse primers (conc. of 10 pmol in 1 μL) and 2 μL of genomic DNAas a template (50 ng μL^−1^) in it. Amplification of *Chit1* was done by initial denaturation at 95 °C for a minute, repeated 35 cycles for denaturation at 95 °C for a minute as the annealing temperatures were followed by denaturation time. The annealing temperature was 45°Cfor *T. longibrachiatum* for a minute*.* The extension was performed at 72 °C for 2 min, and the final extension was at 72 °C for 10 min followed by the final hold at 4 °C.

### Cloning in pGEM T-easy vector

The final PCR products were purified from the gel according to the protocol of the Thermo Scientific Genomic purification kit (Catalog number: K0721). The products of PCR were then ligated into cloning vector pGEM T-easy and then it is transformed into the DH5α strain of *Escherichia coli* (*E. coli*). After a successful transformation, the plasmid was cloned, isolated, and further confirmed before sequencing through restriction using the restriction enzyme *EcoRI*.

### Sequencing and homology analysis

The plasmid was sent to ETON BIOSCIENCES, San Diego, CA, USA, with both M13 forward and reverse primers for sequencing. Sequences for both forward and reverse strands were amassed with DNA SEQMAN PRO's help (https://www.dnastar.com/t-seqmanpro.aspx). The sequence of the vector was then restricted from both ends. The final sequence was then blasted in NCBI for the nucleotides database.

### Characterisation of the chitinase gene

The chitinase gene was characterised before expression in plants. The sequences were blasted in the NCBI database, and their percentage homology and evolutionary relationships were premeditated. For comparison, BLAST analysis^37^ (NCBI; http://www.ncbi/BLAST) was performed to compare the sequence of *Chit1* with releavantchitinases already accessible in databases. The open reading frames (ORFs) for the gene were scrutinised to find out the conserved domain with the help of the Conserved Domain Search Service (CD-Searchhttps://www.ncbi.nlm.nih.gov/Structure/cdd/wrpsb.cgi) tool from NCBI^38^. The overall signal peptide and molecular weight of isolated chitinase was anticipated with software SIGNAL P 3.0^39^ (http://www.cbs.dtu.dk/services/SignalP).

### Expression of chitinase (ORF) in cotton plants

#### Virus-induced gene silencing vector

The vector derived from the Cotton leaf crumple virus (CLCrV) was taken from Brown’s lab, University of Arizona, Tucson, AZ, USA. It was a permanent inventory of clones that were already in action to develop cotton plants with transient gene expression^40^. The ORF coding coat protein of cotton crumple virus (pJRTCLCrVA. 008) was altered with multiple cloning sites (MCS) (Gene bank EU541443) in the Virus-induced gene silencing** (**VIGS) vector. At the same time, the other component of CLCrV that is B, is also taken from Brown’s lab.

### Isolation of chitinase ORF

The open reading frame (ORF) having catalytic domains was isolated from the plasmid. Different primers were designed while keeping information about the restriction sites. Each primer is designed with *EcoRI* in the forward primer and the *NheI* restriction site in the reverse primer. ORFs from *T. longibrachiatum* was amplified from previously cloned chitinases in the pGEM T-easy cloning vector by using *Tri*_ORF_F (5′ATGAGCTCCTTCGTGCTGGGGTC3′and *Tri*_ORF_R (5′GAACTAGCATCTGTAATTTTG3′) primers. Employed PCR conditions were as; initial denaturation at 95 °C for 1 min, the second denaturation was at 95 °C for 1 min with repeated 35 cycles followed by annealing temperatures used according to the ORFs for *T. longibrachiatum.* The annealing temperature was 56 °C for the *Chit1* gene of *T. longibrachiatum.* The time for extension was 2 min at 72 °C with a final extension at 72 oC for 10 min.

### Ligation and construction of VIGS-Chit plasmids

The open reading frame of *T. longibrachiatum* were ligated into the pGEM T-easy vector, and then it was transformed in syrain DH5α of *E. coli*. Plasmids were isolated, sequenced and analysed before their cloning. VIGS vector (CLCrV-A) and plasmids were restricted with *NheI* and *EcoRI* restriction enzymes to clone into the VIGS vector. The ligation was performed at the ratio of 3:1 for both (insert: vector). The restriction analysis was performed for isolation and sequencing of *VIGS-Chit* recombinant plasmids.

### Particle bombardment/ biolistic bombardment

Cultivated cotton seeds (*Gossypium hirsutum *L.*)* were collected from the Centre for excellence in Molecular Biology (CEMB), University of the Punjab and placed in pots that had potting soil under control conditions. All methods were carried out in accordance with relevant guidelines. Five seeds were used in each pot, and pots were vigilantly observed until the first true seedlings leaves emerged after 10–15 days. The experiment was divided into five replicates, and each replicate was representing a pot with five seedlings. All of the seedling’s leaves that emerged inoculated biolistically and then were shifted to new individual pots. These pots were placed in a growth chamber with a light period of 15/9 h at 890 μmol m^−2^ s^−1^ and temperature of about 23–25 °C. The recommended fertiliser MIRACLE-GRO (Miracle Gro Products, Inc.), was used twice or thrice in a week^38^ (Anwar et al.). Different temperature ranges as 30 °C/26 °C or 22 °C/18 °C day/night temperatures were set in IAGS, University of the Punjab, with relative humidity (R.H) of 40% to 50% to analyse the growth of seedlings. The plants were individually grown in styrofoam cups with different potting mixtures as 1/3 peat –lite and 2/3 pea gravel and were transferred to 1500 mL pots. Different fertilisers, weak Hoagland solution and water were applied three to four times to all pots. The micro-projectiles made up of gold with 1.5- μm diameter (inBio) coated with a 10 μg mixture of component A having *Chit1* ORFs and component B having CLCrV were used for bombardment^41^. DNA- coated projectiles were loaded with a filter end of Millipore Swinnex and each leaflet was placed below the outlet and bombarded one time from the distance of 6 cm aprroximately. Almost three to four seedlings were bombarded simultaneously with an external pressure of 40–60 psi. For inoculation of seedlings, a commercially available PARTICLE DELIVERY SYSTEM (BioRad PDS1000-He) was used. It has an infection rate of 80%. Both components (A and B) without *Chit1* gene were used as mock plants.

### Analysis of transgenic expression

The total RNA from transformed cotton plants was extracted by using a QIAGEN kit (catalogue # 74106)^41^. PCR was performed at 20 °C for 15 min, 37 °C for 120 min, and 10 min at 80 °C followed by 35 cycles. The young cotton leaves were taken after two weeks to confirm the transient expression and their total RNA was extracted. cDNA was produced by the “Revert Aid First Strand cDNA Synthesis Kit” (catalogue #K1622, THERMO SCIENTIFIC). The amplification of the coat protein region of CLCrV was performed using both forward GTTCTAGAATCACCTTCCACTATGAGAC and reverse TCAGAATTCCCTTAACGTGCGATAGATTCTGGGGC primers^42^. The final PCR products were run on 1.5% agarose gel for the confirmation of transient expression.

### Chitinase assay

The activity of recombinant chitinase produced by transgenic cotton was evaluated with the help of the Chitinase Assay kit SIGMA-ALDRICH, catalogue # CS0980. The enzymatic hydrolysis of chitinase substrate was determined. It releases p*-*nitrophenol upon hydroxylation, and it can be measured at an absorbance of 405 nm. Almost 1.0 μmol of p-nitrophenol was released from one substrate unit per minute at 37 °C and pH 4.8. The substrates used for endochitinase were 4-Nitrophenyl β-D-N, N0, and N00—triacetylchitriose. The purified chitinase was already provided in the kit, and it was used as a positive control.

### Virulence bioassay of chitinase transgenic plants against *Aphis gossypii*

After estimating and confirming various chitinase enzyme activities in cotton plants through chitinase enzyme assays and VIGS vector, transient cotton plants were allowed to feed by 4th instar nymphs and adults of *A. gossypii.* Data were recorded after regular intervals according to the mortality percentage using modified Abbot’s formula^42^.

### Analysis of data

The final data were analysed and estimated using SPSS, v. 11.5 statistical software (SPSS Inc., Chicago, IL, USA. Before analysis, the percentage data were log transformed.

### Supplementary Information

Supplementary Information.

## Data Availability

The sequence of chitinase gene of *Trichoderma longibrchiatum* was submitted to the GenBank (Accession No. OQ689733) in NCBI under the following link: https://www.ncbi.nlm.nih.gov/nuccore/?term=OQ689733 and also provided in related file.
