# Supplementary material for: Chitinase of Trichoderma longibrachiatum for control of Aphis gossypii in cotton plants
Source: Sci Rep. 2023 Aug 14;13:13181. doi: 10.1038/s41598-023-39965-y (PMC10425378; doi:10.1038/s41598-023-39965-y)
Supplement: Supplementary file 1 — Supplementary Information. [file 41598_2023_39965_MOESM1_ESM.pdf]

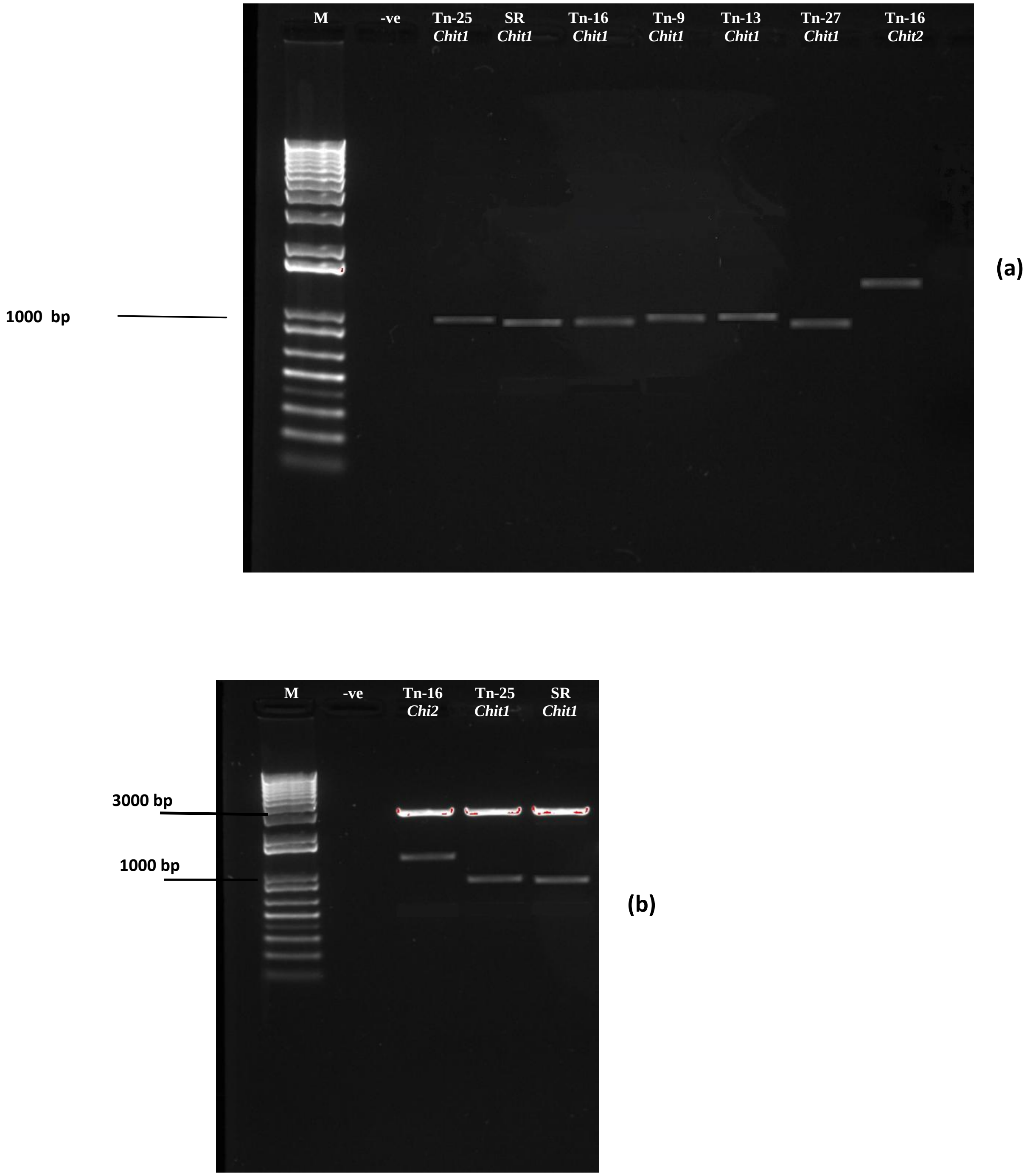

Figure 1: (a) PCR confirmation of partial endochitinase *Chit1* from *T. logibrachiatum* (SR Chit1). M = Promega 1 Kb DNA Plus Ladder. **(b)** Restriction analysis of transformed plasmid by using *EcoR1*restriction enzyme (SR Chit1).

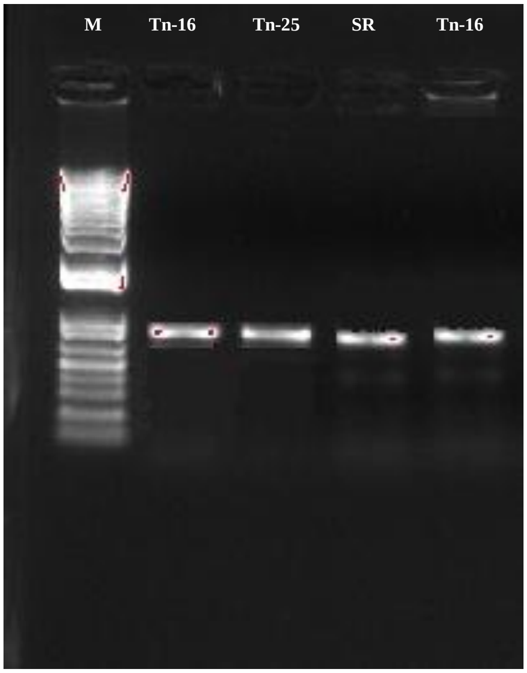

(a)

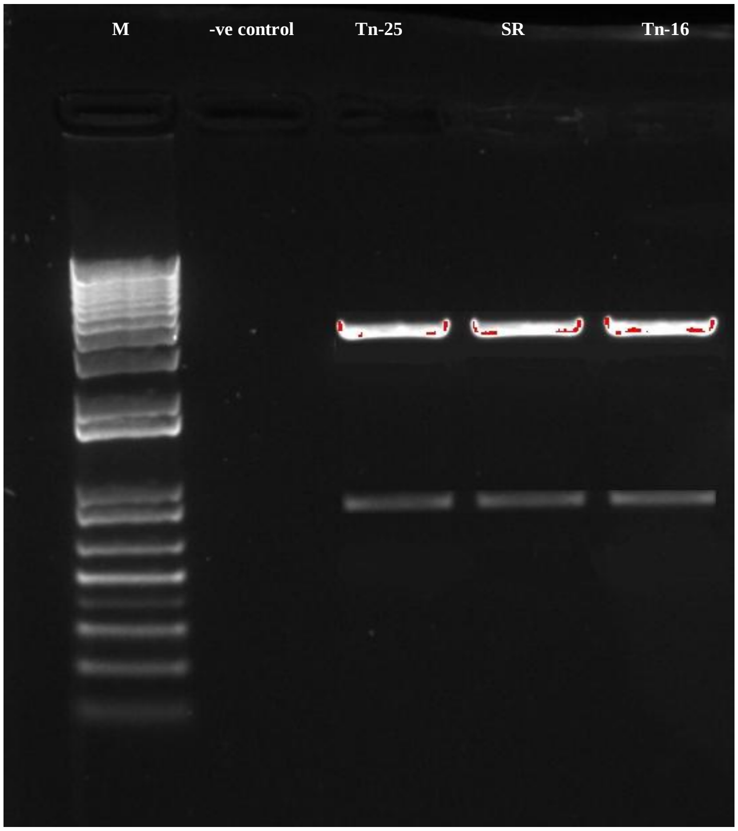

(b)

Figure 4. (a) Amplification of chitinase ORFs from *T. longibrachiatum* (SR) (b) Restriction analysis of *VIGS-Chit* recombinant plasmids (SR). N represents Promega™ 1 kb Plus DNA ladder.
